# Presence of anti-*Toxocara canis* antibodies and risk factors in children from the Amecameca and Chalco regions of México

**DOI:** 10.1186/s12887-015-0385-9

**Published:** 2015-05-30

**Authors:** Nadyeli Nava Cortés, Camilo Romero Núñez, Bautista Gómez Linda Guiliana, Pedro Abel Hernández García, Rafael Heredia Cárdenas

**Affiliations:** Agricultural Sciences and Natural Resources, Amecameca University Center, Autonomous University of the State of México, Km. 2.5 Carretera Amecameca-Ayapango, 56900 Amecameca, México

**Keywords:** *Toxocara*, Zoonotic disease, ELISA, México

## Abstract

**Background:**

Toxocariasis is a zoonotic disease that poses a threat to public health worldwide. This disease primarily affects children and is caused by the presence in the digestive tract of a common roundworm of dogs, *Toxocara canis*, or cats, *Toxocara cati. Toxocara* is responsible for the presentation of various syndromes in humans depending on the affected organs.

**Methods:**

In this study, the prevalence of anti-*T. canis* antibodies was investigated in children aged 3–16 years from semirural populations in the municipalities of Amecameca and Chalco in México. An ELISA was used to determine the presence of anti-*T. canis* antibodies in blood samples.

**Results:**

Of the 183 sera obtained for this study, 22 were positive for anti-*T. canis* antibodies (12.02 %). Of these, 6.50 % were from males and 5.4 % were from females. Risk factors were investigated and it was found that living near a cattle operation had a statistically significant association with (Chi^2^ = 5.51 and *p* = 0.01) and was a risk factor for (OR = 4.25, *p* = 0.02) seropositivity to *T. canis*. Keeping dogs with short hair (Chi^2^ = 3.24 and *p* = 0.07) showed a tendency toward seropositivity for *T. canis*, as did the habit of sleeping with pets (Chi^2^ = 3.46 and *p* = 0.06).

**Conclusions:**

Seropositivity to *T. canis* was confirmed in children in the Amecameca and Chalco regions of México and the risk factors were identified. These findings provide important insight into the prevalence and spread of this zoonotic parasite.

## Background

Dogs are associated with more than 60 zoonotic diseases worldwide, mainly by parasitic organisms, posing a serious health threat to humans [1,2]. Many of these intestinal parasites are eliminated as eggs, larvae or oocysts into the environment, and for this reason, contamination with the feces of dogs in private and public areas such as parks or gardens represents an important source of infection [3,4].

One of the most common infections worldwide caused by parasites is toxocariasis, a zoonotic disease [5] caused by migrating larvae of the roundworm species *Toxocara canis* and *Toxocara cati* [6]. The eggs of this parasitic helminth may be accidentally ingested by humans [7] into the duodenum; once the eggs hatch, they release three-stage larvae (L3) through the action of gastric juice and digestive enzymes, which penetrate the intestinal wall, enter the bloodstream and migrate to different organs, where they lead to syndromes such as visceral larva migrans, covert larva migrans, ocular larva migrans and neurological larva migrans [6,8].

Some risk factors associated with this parasite include: gender, age, socioeconomic status, close contact with domestic animals [9], ingestion of raw meat [7], poor hygiene, inadequate hand washing, nail biting, eating contaminated food, and contact with soil or the hair from cats or dogs contaminated with eggs [4]. Children are the social group most at risk because of their recreation activities, hygiene and close relationship with pets [10].

In México, there have been studies to investigate the prevalence of *T. canis* in recreational areas, and in soil samples and the feces of stray dogs. Rates of 24 % and 67.5 % were obtained [11,12]. The high rate of contamination was thought to reflect the socioeconomic status and sanitation level of the studied region. Similar studies in other countries have also revealed a high prevalence of *T. canis*; for example, 66 % prevalence in Spain and 67 % prevalence in Argentina [13].

Furthermore, seroprevalence studies in children reported rates of 22.22 % in México [14] compared with 86.75 % in Taiwan in 2014 [15]. To investigate the public health threat caused by toxocariasis in more detail in México, in this study, circulating anti-*T. canis* antibodies and risk factors for *T. canis* were investigated in a semirural population in the State of México.

## Methods

### Study population

**Figure 1:**
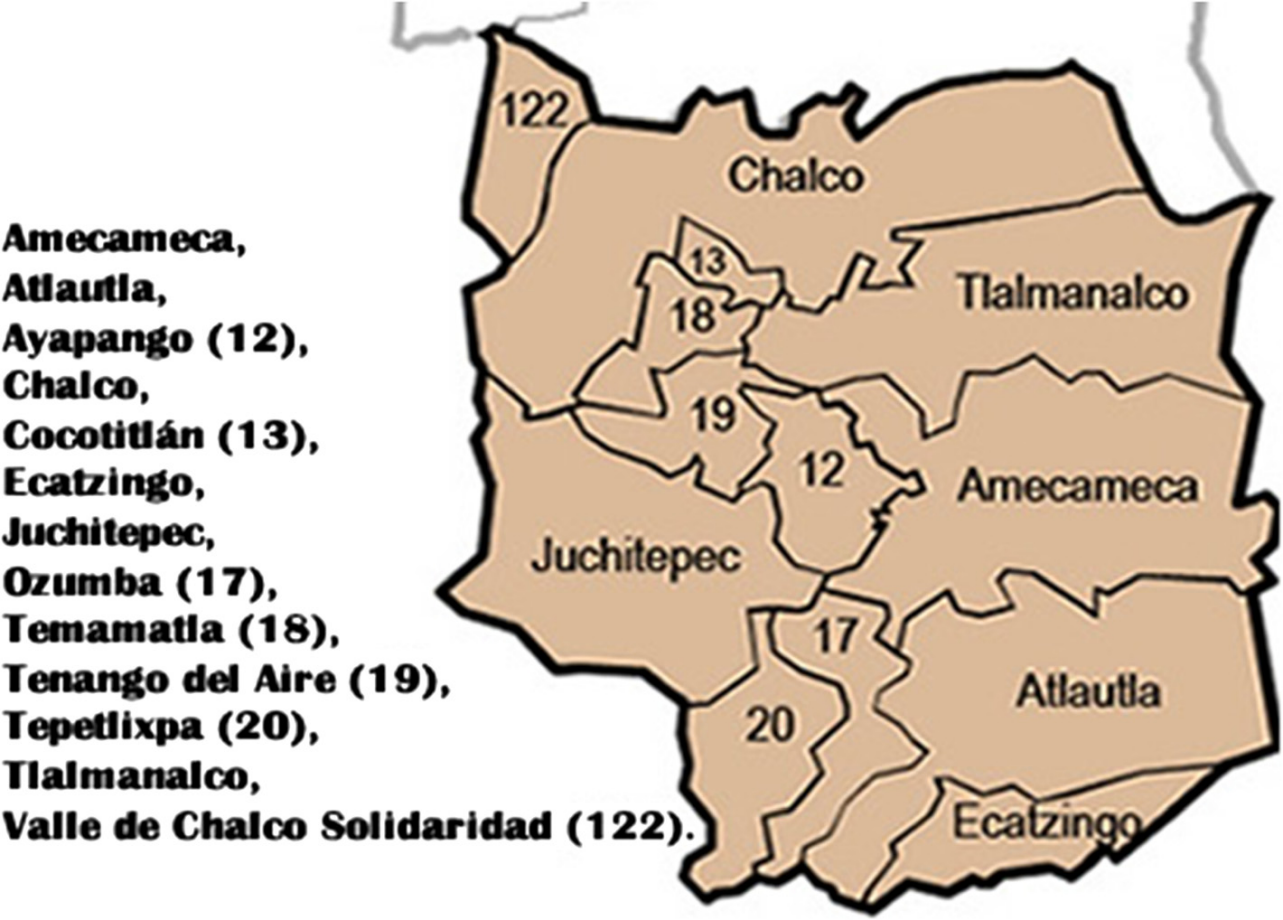
The location of the municipalities of Amecameca and Chalco within the State of México

A total of 183 blood samples were collected from children in the municipalities of Amecameca and Chalco, México (Fig. 1), between April 2013 and February 2014. Of the children sampled, 97 were male and 86 were female, and their ages ranged from 3–16 years. Parents or guardians of minors signed an informed consent form and agreed to participate in the project, which was approved by the ethics committee of the University Center UAEM Amecameca of the Autonomous University of the State of México.

### Epidemiological data collection

Epidemiological data for all of the children included in the study were obtained. These included: age, gender, height, weight, body mass index and background information regarding recent types of pathology – respiratory, dermatological, neurological, allergic and ocular – and the following risk factors: eating habits and hygiene.

### Processing blood samples

A 3-ml sample of blood was obtained from each participant through phlebotomy and these samples were deposited in tubes without anticoagulant and incubated at room temperature for 40 min. Then, samples were subjected to centrifugation at 4000 × *g* for 10 min. The obtained sera were stored at −20 °C according to official Mexican guidelines for the storage of human samples (NOM-003-SSA2-1993).

### Serological testing of blood samples

Serological tests were performed on the blood samples to measure anti-*T. canis* antibodies using a commercial ELISA kit, with a sensitivity of 87.5 % and specificity of 93.3 % (DIAGMEX-*Toxocara*® SA De CV. México). The optical density of the samples was determined using a Microplate Modulus® multiplate spectrophotometer (Turner Biosystems, Kampenhout, Belgium) at a wavelength of 460 nm [16]. The optical density cutoff value was 0.30, and sera with an optical density of 0.30 or greater were considered positive.

### Statistical analysis

To compare the rates of positivity between groups, Fisher’s exact test [16] was applied. The variables obtained were analyzed by the Chi-square test to determine the levels of association; a value of *p* < 0.05 was considered significant. The relative risk of *T. canis* antibodies was calculated from the odds ratio, with a significance of *p* < 0.05. Statistical analysis was performed using JMP® 8.0 software (SAS Institute, Cary, NC, USA).

## Results

Of the 183 sera tested, anti-*T. canis* antibodies were detected in 22 (12.02 %). No statistically significant difference was found between the prevalence of anti-*T. canis* antibodies in males and females (Table 1; *p* = 0.64).

**Table 1:** Comparison of the presence of antibodies with Toxocara canis between genders

Sex	Positive *n* = 22 (%)	Negative *n* = 161 (%)	Total	p*
Female	10 (5.4 %)	76 (41.5 %)	86	0.64
Male	12 (6.5 %)	85 (46.4 %)	97	
Total	12.02 %	87.98 %	183	

*Fisher *p* < 0.05

Regarding risk factors, it was determined that keeping dogs with short hair (Chi^2^ = 3.24 and *p* = 0.07) showed a tendency toward seropositivity for *T. canis*, as did the habit of sleeping with pets (Chi^2^ = 3.46 and *p* = 0.06). By contrast, practicing preventive medicine and coexistence did not show a significant association (Table 2). Living near livestock farms showed a statistically significant association with *T. canis* seropositivity (Chi^2^ = 5.51 and *p* = 0.01) and was also a risk factor for *T. canis* seropositivity (OR = 4.25, *p* = 0.02) (Table 3).

**Table 2:** Association between risk factors and the presence of antibodies against animal-related *Toxocara canis*

Risk factor	Positive *n* = 22	Negative *n* = 161	Chi^2^	*p*	OR	*p*	CI
Pets at home	8	75	0.81	0.36	0.65	0.36	0.26–1.64
Sleeping with pet	0	13	3.46	0.06	0.24	0.33	0.01–4.25
Puppy <6 months	2	17	0.04	0.83	0.84	0.83	0.18–3.94
Dog licks face	2	4	2.66	0.10	3.92	0.12	0.67–22.8
Dewormed >6 months ago (pet)	22	153	2.09	0.14	2.49	0.53	0.13–44.6
Dog with long hair (>1 cm)	3	19	0.25	0.62	1.18	0.80	0.31–4.36
Dog with short hair (<1 cm)	3	50	3.24	0.07	0.35	0.10	0.09–1.23
Contact with dogs or cats outside the home	6	59	0.74	0.38	0.64	0.39	0.24–1.74

*OR* odds ratio, *CI* confidence interval

*p* < 0.05

**Table 3:** Risk factors associated with housing

Risk factor	Positive *n* = 22	Negative *n* = 161	Chi^2^	*p*	OR	*p*	CI
Frequent visits to public parks	9	59	0.15	0.69	1.19	0.69	0.48–2.69
Live near livestock farming	4	8	5.51	0.01	4.25	0.02	1.16–15.5
Garden at home	6	53	0.28	0.59	0.76	0.59	0.28–2.06

*OR* odds ratio, *CI* confidence interval

*p* < 0.05

## Discussion

In Mexicali, México, a previous study analyzing the seroprevalence of *T. canis* in children reported a rate of 10.6 %, less than that in the present study (12.02 %), and a higher percentage of seropositive males (53.1 %) than females, which they attributed to differences in the games played by children and their resulting close contact with the environment [10]. In other research [17], a study conducted in southeastern São Paulo, Brazil, with volunteer donors aged 19–65 years, reported seroprevalence of 8.7 % and concluded that gender (*p* = 0.69) and age (*p* = 0.99) were not associated with parasitoses. These findings were in agreement with the present study in which no significant association was reported between gender and the presence of *T. canis* antibodies (*p* = 0.64). A study by Getaz and colleagues in 2007 found no significant association between seropositivity to *T. canis* and asthma, but did identify an association with nocturnal wheezing [18].

Hygiene habits, housing area and customs are factors that primarily influence the variation of seroprevalence of *T. canis*, and the risk of transmission increases with the degree of environmental pollution. Three research teams [19–21] conducted studies to identify the predisposing factors for the transmission of parasitic zoonoses and determined that pets in the bedroom, not regularly cleaning feces and no or inadequate hand washing after contact with animals posed the greatest risks of transmitting zoonotic diseases. Contamination with *Toxocara* spp. from the land in parks, playgrounds, gardens and beaches in urban and suburban areas is considered a critical factor for toxocariasis, as well as contact with dogs. The results of the current study showed that the potential risk factors of keeping dogs (Chi^2^ = 3.24, *p* = 0.07) and sleeping with dogs (Chi^2^ = 3.46, *p* = 0.06) showed a tendency toward seropositivity for *T. canis* but this was not a significant association. Another study of seroprevalence in children in México [14] found that frequent contact with pets was not associated with the presence of toxocariasis (Chi^2^ = 0.80, *p* = 0.37). A study in Argentina, however, found that contact with animals was an important risk factor for *T. canis* exposure in children (OR = 5.02, *p* = 0.001) [22]. These discrepancies may reflect differences between geographical locations or the size of characteristics of the sample populations between studies. Living near a cattle operation was found in the current study to show a statistically significant association (Chi^2^ = 5.51, *p* = 0.01) with the presence of anti-*T. canis* antibodies in children, potentially linked with the presence of stray dogs on such premises.

## Conclusions

The results of this investigation revealed a seroprevalence of 12.02 % for *T. canis* in children in the municipalities of Amecameca and Chalco, in the State of México. We observed a higher percentage of seropositive males than females, and attributed this to their increased activity in recreational areas. Living near livestock farms was identified as a risk factor for exposure to *T. canis*, as assessed by the presence of anti-*T. canis* antibodies.
